# Creation of localized spins in graphene by ring-opening of epoxy derived hydroxyl

**DOI:** 10.1038/srep26862

**Published:** 2016-05-26

**Authors:** Jie Chen, Weili Zhang, Yuanyuan Sun, Yongping Zheng, Nujiang Tang, Youwei Du

**Affiliations:** 1 Nanjing National Laboratory of Microstructures & Jiangsu Provincial Laboratory for Nanotechnology, Nanjing University, Nanjing 210093, PR China

## Abstract

Creation of high-density localized spins in the basal plane of graphene sheet by introduction of *sp*^3^-type defects is considered to be a potential route for the realization of high-magnetization graphene. Theoretical and experimental studies confirmed that hydroxyl can be an effective *sp*^3^-type candidate for inducing robust magnetic moment. However, the artificial generation of hydroxyl groups for creating high-density spins on the basal plane of graphene sheet is very scarce. Here we demonstrate that high-content hydroxyl groups can be generated on the basal plane of graphene oxide (GO) sheet by ring opening of epoxy groups. We show that by introduction of 10.74 at.% hydroxyl groups, the density of localized spins of GO can be significantly increased from 0.4 to 5.17 *μ*_B_/1000 C. Thus, this study provided an effective method to obtain graphene with high-density localized spins.

Magnetism of graphene is gaining increasing interest in recent years for its potential application in spintronics because its long spin-relaxation time provides the ideal condition to manipulate the spins^1^,^2^,^3^. Introduction of high-density, ferromagnetic coupling spins is crucial for the realization of ferromagnetic graphene with high magnetization for the novel spintronic applications^4^,^5^,^6^,^7^, and this also is of great importance for the wide applications of this kind of light magnetic material. Up to date, creating robust magnetic moments in graphene still keeps a great challenge. There are two approaches of inducing magnetic moments in graphene: creation of the edge magnetic moments at the edge sites by edge-type defects (the edge-type approach) and creation of the basal-plane magnetic moments on the basal-plane sites by *sp*^3^-type defects (the basal-plane approach). Recently, *sp*^3^-type functionalization is widely recognized to be an effective route to generate magnetic moments on the basal-plane sites of graphene sheets^8^,^9^,^10^,^11^,^12^,^13^,^14^,^15^. Because plenty of these *sp*^3^-type defects can be introduced on the basal plane of the graphene sheet, this kind of approach is considered as hitherto the most promising approach to induce high-density localized spins^8^. Typically, *sp*^3^-type approach has been experimentally realized by functionalizing graphene with H and F^8^,^16^,^17^,^18^. Even so, the spin density reported is still low for graphene or its derivative up to now because such adatoms tend to aggregate arising from the low migration barrier^8^. Thus, it is urgent to develop effective *sp*^3^-type approaches to create high-density localized spins on basal plane of graphene sheets.

Graphene oxide (GO) is a typical graphene derivative. To micron-sized GO sheet, there are generally some basal-plane oxygen-containing groups (epoxy and hydroxyl groups) on its basal plane and small fraction of edge oxygen-containing groups (carbonyl and carboxyl groups) at edge sites^19^,^20^. It is greatly different from the case of nano-sized GO quantum dot or GO nanoribbon, wherein edge oxygen-containing groups dominate the oxygen-containing groups. Theoretically, single epoxy group is nonmagnetic because it creates equal defects in A and B sublattice^3^. Nevertheless, both theoretical and experimental results showed that single hydroxyl group can effectively induce localized spin on the basal plane of graphene sheet^11^,^14^,^15^,^21^,^22^,^23^, and this makes hydroxyl group to be used as a *sp*^3^-type candidate for inducing localized spins on the basal plane of graphene sheet. Even the superiority of hydroxyl group in inducing localized spin, the artificial generation of hydroxyl groups for creating high-density spins on the basal plane of graphene sheet is very scarce.

In general, micro-sized GO sheet obtained by chemical exfoliation of graphite powder has high-content epoxy groups, which is much more than that hydroxyl groups^20^,^22^,^23^,^24^,^25^,^26^. Recent research showed that epoxy group on the basal plane of GO sheet may convert to hydroxyl groups via ring opening in alkaline condition^26^. The high-content hydroxyl groups have been effectively generated by treating GO using sodium borohydride (NaBH_4_) as the alkaline reagent^27^,^28^,^29^,^30^. Reasonably, this may afford the feasibility for creating high-density spins on basal plane of GO sheets by hydroxyl groups generated via ring opening of epoxy groups. In this study, hydroxylated graphene (OHG) with high-content hydroxyl groups was obtained by treating GO using NaBH_4_. Our results clearly indicate that by generating high-content hydroxyl groups via ring opening of epoxy groups, the high-density localized spins can be created on the basal plane of OHG sheets. Moreover, we show that the magnetization of GO can be increased from 0.136 to 3.11 emu/g via ring opening of epoxy groups combined Ar annealing method.

## Results

### Microstructures of GO and OHG

**Figure 1:**
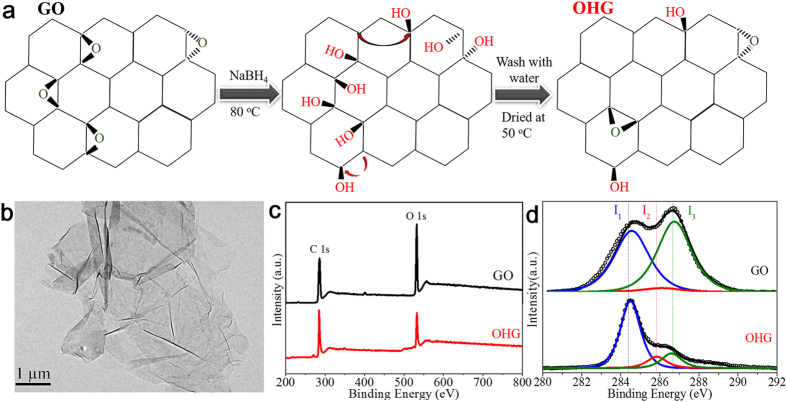
Microstructures of GO and OHG. (**a**) Schematic illustration for the creation of hydroxyl groups by ring opening of epoxy groups. (**b**) TEM image of OHG. (**c**) XPS of GO and OHG over a wide range of binding energies (0–1000 eV). (**d**) Fine-scanned C 1s spectra of GO and OHG. The black hollow circles and the color lines are measured dots and fitting curves.

We synthesized GO by chemical exfoliation of natural flake graphite powder. It is found that GO is of several micrometers in size (see Supplementary Fig. S1a) and is few-layered (see Supplementary Fig. S1b). It has been demonstrated that the epoxy groups can be converted to hydroxyl groups by ring opening of epoxy groups in alkaline condition^26^, and the mechanism of the creation of hydroxyl groups by ring opening of epoxy groups can be illustrated in Fig. 1a. Thus, it is reasonable to speculate that in alkaline NaBH_4_ solution, a large amount of hydroxyl groups can be generated on the basal plane of GO sheets. Shown in Fig. 1b is typical transmission electron microscopy (TEM) image of OHG. As found, OHG maintains the two-dimension ultrathin flexible structure with the size of several micrometers. To detect the content of epoxy and hydroxyl groups, X-ray photoemission spectrum (XPS) measurements were carried out. Shown in Fig. 1c is the XPS spectra of GO and OHG over a wide range of binding energies. It is found that compared to the case of GO, there is a clear decrease in the O 1s peak in OHG, indicating that GO was reduced by NaBH_4_, similar to the result reported^26^. Figure 1d shows the Fine-scanned C 1s spectra of GO and OHG. As known, the clear peak I_1_ (284.5 eV), I_2_ (285.8 ± 0.2 eV) and I_3_ (286.5 ± 0.2 eV) respectively correspond to *sp*^*2*^ carbon atoms with their first nearest neighbors being also graphitic *sp*^*2*^ C atoms, hydroxyl group and epoxy group^22^,^26^,^31^. In the case of GO, the contents of epoxy and hydroxyl groups are ~30.41 (defined as 100 epoxy groups/C at.%) and 3.51 at.% (defined as 100 hydroxyl groups/C at.%), implying that epoxy groups are the mainly oxygen-containing groups on the basal plane of GO sheets. It is known that there is difference in the content and distribution of hydroxyl and epoxy groups in GO reported because of the various synthesis conditions^22^,^23^,^25^,^32^,^33^,^34^, and this is considered to be the reason that the reported magnetism of GO via chemical exfoliation of graphite is multifarious^22^,^23^. After the NaBH_4_ reduction, the content of epoxy groups decreased to only 8.08 at.% synchronized with the significant increase in the content of hydroxyl groups (10.74 at.%, Table 1). As illustrated in Fig. 1a, the ring opening of single epoxy group can result in the generation of two hydroyl groups. However, the decomposition of ca. 23.71 at.% epoxy groups in GO resulted in the unexpectable increase of only ca. 8 at.% in the content of hydroyl groups in OHG, implying that the generation efficiency of hydroxyl groups via ring opening of epoxy groups is low. As reported^26^, the two neighbored hydroyl groups (diol groups, which emerged by ring opening of epoxy group) is unstable which may be removed or reformed during the alkaline treatment at 80 °C. This may contribute to the low generation efficiency of hydroxyl groups via ring opening of epoxy groups. However, the density functional theory calculation showed that hydroxyl group attached within an aromatic domain is unstable, and can migrate on the basal plane of graphene sheets because of its low migration barrier^35^. Therefore, two adjacent hydroxyl groups will migrate and become nonadjacent during the alkaline treatment at 80 °C and/or the following drying process and, thus survive on the basal plane of OHG sheets^14^,^15^. Actually, the fact that the high-content hydroxyl groups have been generated on the basal plane of OHG sheets just suggests the migration of diol groups and thus the appearance of the stable and nonadjacent hydroxyl groups. Clearly, our results indicate that high-content nonadjacent hydroxyl groups have been generated on the basal plane of GO sheets by ring opening of epoxy groups.

### Magnetic properties of GO and OHG

**Figure 2:**
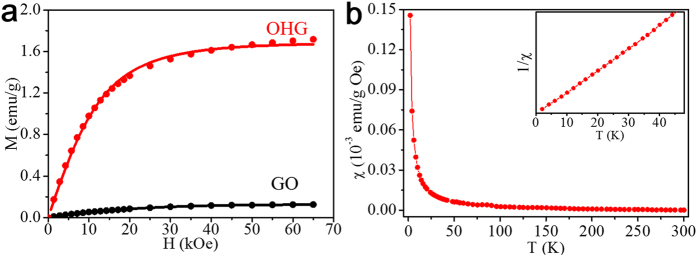
Magnetic properties of GO and OHG measured by a superconducting quantum interference device (SQUID) magnetometer. (**a**) *M-H* curves at measured 2 K. Symbols are the measurements and solid lines are fit to Brillouin function. (**b**) *χ-T* curve of OHG measured from 2 to 300 K under the applied field *H* = 1 kOe. Inset: the *1/χ–T* curve from 2 to 40 K.

We then performed the magnetic measurements of GO and OHG. Figure 2a shows the 2 K mass magnetization *M* (after subtracting the corresponding linear diamagnetic characteristic) as a function of field *H* of GO and OHG. The *M*-*H* curves of both GO and OHG are well fitted using the Brillouin function


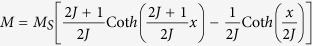


where saturation magnetization *M*_*s*_* = NgJμ*_B_, *x = gJμ*_B_*/k*_B_*T*, *g* is the *g*-factor (assuming *g* = 2), *J* is the angular momentum number, *N* is the number of spins and *k*_B_ is the Boltzmann constant. *M*_*s*_ at 2 K can be obtained by fitting the corresponding *M*-*H* curves. It is found that *M*_*s*_ of GO is low (0.136 emu/g). By contrast, the value of OHG is as high as 1.71 emu/g, 13.6 times higher than that of GO. Subsequently, the spin density of GO and OHG can be extracted from the corresponding *M*_*s*_, which was summarized in Table 1. As found, the spin density of OHG is high up to 5.17 *μ*_B_/1000 C, 12.9 times higher than the value of GO (0.4 *μ*_B_/1000 C). It is clear that the spin density of GO has been significantly increased after introduction of high-content hydroxyl groups via ring opening of epoxy groups. In additional, different from GO which shows the fitted *J* value of 0.9, OHG shows the high fitted *J* value of 1.8. It suggests the formation of the larger spin clusters in OHG, similar to the theoretical results on large spin clusters generated by hydroxyl clusters^11^,^23^. The higher *J* value in OHG implies that the spin clusters grew after introduction of high-content hydroxyl groups via ring opening of epoxy groups. Figure 2b shows the *χ-T* curve and corresponding *1/χ–T* curve (inset in Fig. 2b) of OHG. The purely Curie-like paramagnetic behavior of OHG is corroborated by fitting of *χ*(*T*) curve to the Curie law *χ = NJ*(*J* + 1)*g*^2^*μ*_B_^2^/3*k*_B_*T*. In other words, the creation of hydroxyl groups results in the enhancement of the paramagnetism of GO.

## Discussion

**Figure 3:**
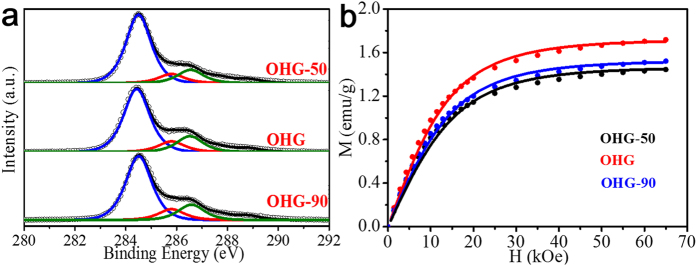
Microstructures and magnetic properties of the OHG samples. (**a**) Fine-scanned C 1s spectra of the OHG samples. The black hollow circles and the color lines are measured dots and fitting curves. (**b**) *M-H* curves of the OHG samples measured at 2 K by SQUID. Symbols are the measurements and solid lines are fit to Brillouin function.

To investigate the effect of the dosage of NaBH_4_ on the ring opening of epoxy groups and magnetic properties, two extra OHG samples (OHG-50 and OHG-90) were prepared by changing the dosage of NaBH_4_ (50 and 90 mM). As illustrated in Fig. 3a and Table 1, it is found that after reduction, (i) all the OHG samples have lower content of epoxy groups but higher content of hydroxyl groups than GO; and (ii) with the increase of NaBH_4_ dosage, the content of hydroxyl groups increases. It shows that the content of hydroxyl groups in GO can be tuned in a relatively wide range by adjusting the usage of NaBH_4_. Table 1 also gives the spin density of all the OHG samples extracted from the corresponding *M*_*s*_ measured at 2 K (Fig. 3b). Compared to GO, all of the three OHG samples show the clear increase in *M*_*s*_ and spin density.

Additionally, we can see that compared with OHG, OHG-90 has the higher content of hydroxyl groups but the lower magnetization. This may results from the various forms of hydroxyl groups on the basal plane of GO sheets. Theoretical calculations showed that single hydroxyl group can induce ~1 *μ*_B_ due to the localized *sp*^*3*^ structure^11^,^21^,^36^. Reasonably, the pattern or specific distribution of hydroxyl groups may play a key role in their magnetism-inducing efficiency, similar to the case of fluorine on graphene sheet^8^,^17^. Especially, the residual epoxy groups on the basal plane of GO sheets can inevitably affect the distribution and its magnetism-inducing efficiency of hydroxyl groups may constraint the magnetism. Moreover, the migration of hydroxyl groups during the delamination of graphite can theoretically lead to the formation of large hydroxyl clusters on the basal plane of the graphene sheet which can induce large spin clusters^11^,^14^,^15^,^23^. Generally, *J* is reported as 0.5, 1 or 2.5 in GO which corresponds to magnetically coupled unpaired electrons^8^,^22^,^23^,^37^,^38^. The difference in the *J* values in the OHG samples implies that the spin clusters formed are various, which indicates that the formation of the hydroxyl clusters is complex.

**Figure 4:**
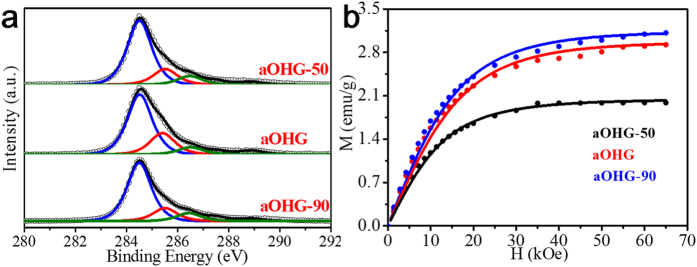
Microstructures and magnetic properties of the aOHG samples. (**a**) Fine-scanned C 1s spectra of the aOHG samples. The black hollow circles and the color lines are measured dots and fitting curves. (**b**) *M-H* curves of the aOHG samples measured at 2 K by SQUID. Symbols are the measurements and solid lines are fit to Brillouin function.

Previous experiment has demonstrated that (i) by annealing of GO at an appropriate temperature ranged from 300 to 500 °C, some of the epoxy groups can be removed and extra hydroxyl groups can be generated, and (ii) the *M*_*s*_ and spin density can be increased^23^. Wherein, the content of hydroxyl groups of the annealed GO remains still low, which limits its magnetization and spin density. By annealing the OHG samples at 300 °C, we obtained the annealed OHG (aOHG) samples. It is found that after annealing of OHG, the content of hydroxyl groups increases further (Fig. 4a and Table 2). As shown, the content of hydroxyl groups in aOHG can reach high up to ca. 14.94 at.%, which is much higher than the value reported^23^. Or say, some extra hydroxyl groups were generated by the decomposition of the residual epoxy groups during annealing. However, compared to OHG-90, aOHG-90 shows the unexpectable decrease in the content of hydroxyl groups. As mentioned above, annealing will cause the migration of hydroxyl groups because of the relatively low migration barrier. As a consequence, two nonadjacent hydroxyl groups may become two adjacent hydroxyl groups, which will result in the disappearance of the two nonadjacent hydroxyl groups. Therefore, the decrease in the content of hydroxyl groups in aOHG-90 may attribute to its excessive hydroxyl groups on the basal plane of OHG-90 sheets, which may provoke the transformation from nonadjacent hydroxyl groups to adjacent hydroxyl groups and, thus decrease the content of hydroxyl groups.

The magnetic properties of the aOHG samples were measured, and their spin densities extracted from the corresponding 2 K *M*_*s*_ (Fig. 4b) are summarized in Table 2. It is found that compared to the OHG samples, all of the corresponding aOHG samples show a clear increase in the spin density and *M*_s_. The spin density of aOHG-90 is high up to 8.72 *μ*_B_/1000 C, much higher than the values of other graphene and its derivatives reported (see Supplementary Table S1). In additional, there is difference in the *J* values between OHG and the corresponding aOHG. It implies that the spin clusters undergo complex transformation, which may attribute to the migration and reformation of hydroxyl groups during annealing. Anyway, the generation of the high-content hydroxyl groups via ring opening of epoxy groups combined annealing affords a chance to tune the density of spin clusters. We can consider that this will facilitate the realization of high-magnetization graphene by altering the distribution of hydroxyl clusters.

In summary, the high-content hydroxyl groups were generated on the basal plane of GO sheets through a ring opening of epoxy groups. The magnetic results showed that the spin density of GO can reach high up to 5.17 *μ*_B_/1000 C by introduction of high-content hydroxyl groups. Therefore, the study reported an effective way to obtain graphene with high-density spins and, thus provided a potential method for the realization of high-magnetization graphene.

## Methods

### Preparation of GO, the OHG samples and the aOHG samples

GO was synthesized by chemical exfoliation of natural flake graphite powder^22^. To prepare OHG, 70 mM of NaBH_4_ was added into the GO dispersion after pH being adjusted to 9~10 with the addition of 5 wt.% sodium carbonate solution. The mixture was then kept at 80 °C for 1 h under constant stirring. Thereafter, the dispersion turned from dark brown to black accompanied by outgassing. The mixture was washed with deionized water for 10 times until the PH reached to 7. After dried in a vacuum oven at 50 °C, OHG was obtained. To prepare the aOHG samples, all the OHG samples were annealed at 300 °C in argon for 1 h. One can see the color of different samples during the reduction (see Supplementary Fig. S2).

### Characterization

The morphologies of the samples were investigated by TEM (model JEM–2100, Japan). XPS measurements were performed on PHI-5000 VersaProbe using Al Ka radiation. The magnetic properties of the samples were measured by SQUID magnetometer with sensitivity less than 10^−8 ^emu (MPMS-XL, USA). The 3d impurity elements of all the samples were measured by inductively coupled plasma spectrometry (Jarrell-Ash, USA), and are negligible (<50 ppm, see Supplementary Tables S2 and S3).

## Additional Information

**How to cite this article**: Chen, J. *et al.* Creation of localized spins in graphene by ring-opening of epoxy derived hydroxyl. *Sci. Rep.* **6**, 26862; doi: 10.1038/srep26862 (2016).

## Supplementary Material

Supplementary Information

## Figures and Tables

**Table 1:** The fitted values of saturated magnetization *M*_*s*_, spin density and spin angular momentum number *J* of GO and the OHG samples by using the Brillouin function.

Samples	GO	OHG-50	OHG	OHG-90
Epoxy groups (at.%)	30.41	6.7	8.08	7.81
Hydroxyl groups (at.%)	3.51	9.11	10.74	13.1
*M*_*s*_ (emu/g)	0.13	1.44	1.71	1.52
Spin density (*μ*_B_/1000 C)	0.4	4.27	5.17	4.65
*J*	0.9	1.5	1.8	1.7

**Table 2:** The fitted values of saturated magnetization *M*_*s*_, spin density and spin angular momentum number *J* of the aOHG samples by using the Brillouin function.

Samples	aOHG-50	aOHG	aOHG-90
Epoxy groups (at.%)	3.91	2.38	3.58
Hydroxyl groups (at.%)	14.02	14.94	11.62
*M*_s_ (emu/g)	1.98	2.92	3.11
Spin density (*μ*_B_/1000 C)	5.75	8.24	8.72
*J*	2.0	1.4	1.5
