# Supplementary material for: Creation of localized spins in graphene by ring-opening of epoxy derived hydroxyl
Source: Sci Rep. 2016 May 26;6:26862. doi: 10.1038/srep26862 (PMC4881012; doi:10.1038/srep26862)
Supplement: Supplementary Information [file srep26862-s1.pdf]

Supplementary Information

Creation of localized spins in graphene by ring-opening of epoxy derived hydroxyl

Jie Chen, Weili Zhang, Yuanyuan Sun, Yongping Zheng, Nujiang Tang & Youwei Du

Nanjing National Laboratory of Microstructures & Jiangsu Provincial Laboratory for Nanotechnology, Nanjing University, Nanjing 210093, China.

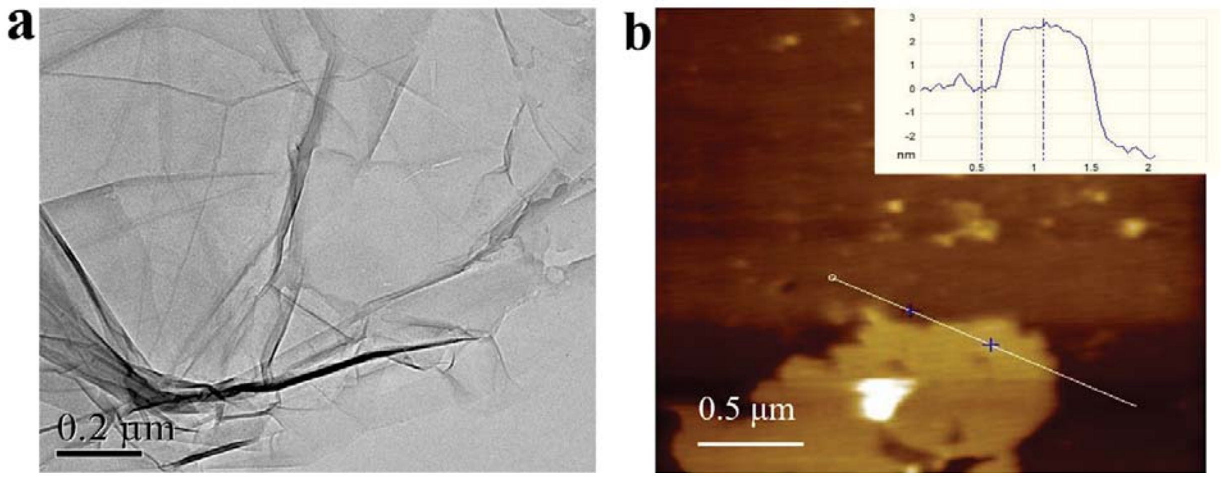

Supplementary Figure S1 | Microstructure of GO. (**a**) TEM image. (**b**) Tapping mode AFM height image. Inset: The corresponding height profiles of the white lines with blue crosses. The blue lines correspond to the blue crosses.

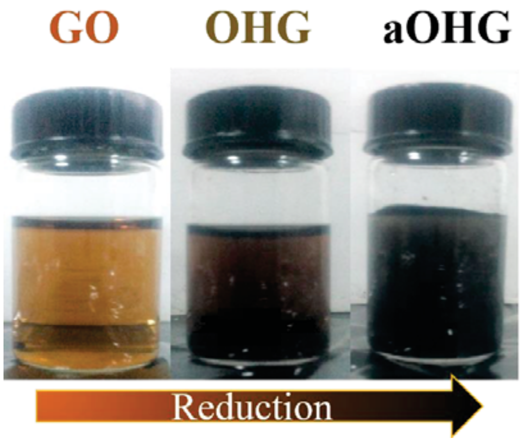

Supplementary Figure S2 | Photograph of GO, OHG and aOHG. One can see the color of different samples during the reduction.

Supplementary Table S1 | Comparison of magnetic properties of OHG and other graphene and its derivatives reported.

Graphene and its derivatives	M_s (emu/g)	Spin density (μ_B / 1000 C)	References
This work	~3.1	9	This work
Hydroxylated graphene	~2.4	6 μ_B / 1000 C	*Sci. Rep.* **5**, 8448 (2015).
Graphene laminate	~ 0.1	1 μ_B / 20000 C	*Phys. Rev. Lett.* **105**, 207205 (2010)
Fluorinated graphene	~ 0.2	1 μ_B / 1000 C	*Nat. Phys.* **8**, 199 (2012)
Fluorinated reduced graphene oxide	~ 0.8	3 μ_B / 1000 C	*ACS Nano* **7**, 6729 (2013)
H-doped graphene	0.006	Not mentioned	*ACS Nano* **7**, 5930 (2013)
C^{4+} / H^{+}-irridiated graphene	< 0.02	Not mentioned	*Nat. Phy.* **8**, 199 (2012)
Unzipped graphene nanoribbon	~ 0.25	Not mentioned	*Nano Lett.* **12**, 1210 (2012)
Reduced graphene oxide	0.02	Not mentioned	*Nano Lett.* **9**, 220 (2009)

Supplementary Table S2 | The contents of the metal impurities of GO and the OHG samples measured by ICP spectrometry. The unit is 'ppm', and 'ND' denotes 'not found'.

Samples	GO	OHG-50	OHG-70	OHG-90
Fe	11.4	11.6	13.2	11.3
Mn	13.7	32.6	26.9	23.3
Ni	ND	ND	ND	ND
Co	ND	ND	ND	ND
Cr	ND	ND	ND	ND
Zn	ND	ND	ND	ND

Supplementary Table S3 | The contents of the metal impurities of the aOHG samples measured by ICP spectrometry. The unit is ‘ppm’, and ‘ND’ denotes ‘not found’.

samples	aOHG-50	aOHG-70	aOHG-90
Fe	15.6	23.2	10.3
Mn	14.7	16.7	23.4
Ni	ND	ND	ND
Co	ND	ND	ND
Cr	ND	ND	ND
Zn	ND	ND	ND
